# Aviator’s Fracture - Bilateral Fracture-Dislocation of Talus in a 29-year-old Patient: A Case Report

**DOI:** 10.5704/MOJ.1911.015

**Published:** 2019-11

**Authors:** G Noia, N Silluzio, G Sircana, G Maccauro, A Ziranu

**Affiliations:** Department of Orthopaedics and Traumatology, Fondazione Policlinico Universitario A. Gemelli IRCSS, Rome, Italy; * Department of Orthopaedics and Traumatology, University of Messina, Messina, Italy

**Keywords:** aviator, bilateral fracture dislocation, talus

## Abstract

Bilateral fracture-dislocation of the talus is a rare occurrence. It represents 0.06% of the dislocations and 2% of the traumas of the talus. We report the case of a 29-year-old patient with an exposed bilateral fracture of the talus following a plane accident. On the right ankle, the patient had a fracture-dislocation Hawkin 3 Gustilo II, on the left ankle presented a Hawkin 4 Gustilo IIIB. The patient was treated within six hours from the trauma. We reduced the dislocation and performed an osteotomy of the tibial malleolus and osteosynthesis of the fracture with screws. The definitive stabilisation has been achieved in both limbs with an external fixator. We evaluated the patient at 1, 3, 6, 8, 12 and 18 months from treatment, with a radiograph and with SF-36 and Foot and Ankle Disability Index questionnaires. No infection was reported, radiographs showed a successful consolidation of the fracture in both limbs. At the one year follow-up, the patient was able to walk without aids and there were no signs of osteonecrosis on the MRI. The treatment of these lesions requires timely treatment, an anatomical reduction of the fracture and patient's collaboration. The use of external fixator with internal osteosynthesis represents a good therapeutic option in Hawkins 3 and 4 type fractures.

## Introduction

Fractures of the talus are usually the consequence of violent trauma, typically occurring in traffic accidents and falls from height (e.g. sudden landings). The mechanism of injury is a compression of the talus with forces transmitted from the tibia (weight force) and the ground. These fractures rarely occur; they represent 0.5% of all fractures and 13% of these lesions are open^1^. Avascular necrosis is the most frequent complication in fractures of the body and neck of the talus, due to the peculiar vascularisation of the talus. The blood supply of the neck of talus comes from the anterior tibial artery that runs across the superior talus-scaphoid ligament. For these anatomical reasons, there is a high chance of avascular necrosis of the neck of the talus^2^. These fractures require timely treatment, anatomical reduction of the fracture and patient's collaboration.

## Case Report

**Fig. 1:**
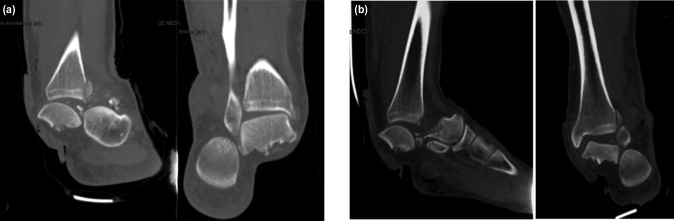
(a) and (b) Pre-operative TC section of both ankles.

**Fig. 2:**
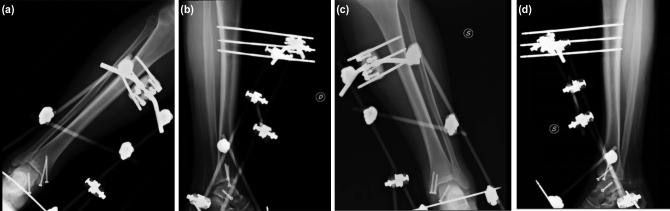
(a), (b), (c) and (d) Post-operative radiograph, show the anatomical reduction of both ankles.

We report the case of a 29-year-old patient (flight instructor) with an exposed bilateral fracture-dislocation of the talus following a plane accident. The impact of the anteroinferior part of the aircraft determined a dorsiflexion and eversion trauma of both feet. In the emergency room, 2g of ceftriaxone was administered intravenously and repeated after 24 hours. On the right ankle, the patient had a medial wound of about 5cm with type II exposure according to Gustilo-Anderson classification, while on the left ankle a 7cm wound with IIIB Gustilo-Anderson exposure was reported. A radiograph demonstrated a bilateral exposed fracture-dislocation of the talus. A CT-scan of both ankles was performed, showing a bilateral fracture-dislocation of the talus classified as Hawkin type III on the right ankle and Hawkin IV on the left ankle (Fig. 1). The patient underwent surgery within six hours from the trauma. We performed a wound debridement and lavage with 3L of saline solution. An osteotomy of the tibial malleolus was performed to gain access to the tibio-talar joint. Then, reduction of the dislocation was obtained and osteosynthesis of the talus fractures was achieved with 4.5mm cannulated screws. Two screws were used for the stabilisation of the osteotomy on each side. The definitive stabilisation was obtained in both limbs with an external fixator (EF) (Fig. 2).

**Fig. 3:**
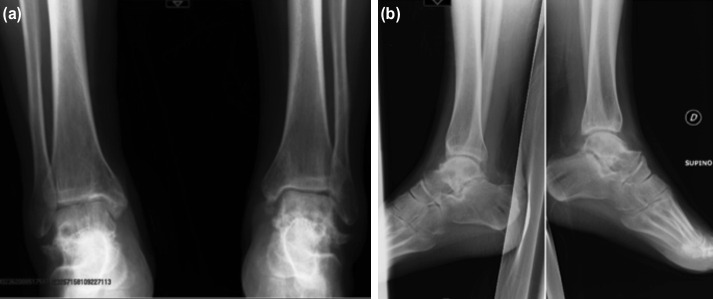
(a), (b) At 18 months of FU, radiograph after removal of the screws. Radiograph shows a severe bilateral talonavicular arthrosis and moderate arthrosis of the sub-talus joint. The patient was satisfied with his quality of life and he returned to fly with his aeroplane.

The patient was re-evaluated one, 3, 6, 8, 12 and 18 months after surgical treatment with radiological and clinical examinations with a radiograph and the administration of SF-36 and Foot and Ankle Disability Index (FADI) questionnaire. Two months after trauma, the EF was removed and the ankle was immobilised in a walker boot. Six months after surgical treatment, weight-bearing was allowed with crutches and walker boot. Afterwards, walker boot was removed and a bivalve-type brace was recommended. At the six months follow-up, the radiograph did not show a Hawkin’s sign. Eight months after the trauma, no infection was reported and the radiograph showed a successful consolidation of the fracture in both limbs. The FADI score one month after surgery was 14.4, three months after surgery 31.7, six months after surgery 69.2, one year after surgery 86.5. One year after trauma, the patient was able to walk without aids and there were no signs of osteonecrosis on the MRI. The patient-reported quality of life was assessed with the SF-36 score, showing good quality of life. A slight deficit (Force=4) of the left extensor hallucis longus remained. One year MRI was compared with the previous MRI, detecting no significant changes in bone and a reduction of the oedema of the soft tissues. After 18 months of follow-up, the patient was considered eligible for the removal of the screws. The post-operative radiograph showed a severe bilateral talo-navicular osteoarthritis, moderate osteoarthritis of the sub-talar joint and talar subcondral sclerosis, unchanged compared to previous radiographs (Fig.3). SF-36 score did not change from the one-year evaluation, the patient was satisfied with his life quality and returned to fly. Considering the radiograph unmodified appearance and the presence of no symptoms, no further radiological imaging was requested.

## Discussion

This type of fracture represents, according to various data of the literature, a rare occurrence and is not free from various complications, the most frequent being the avascular osteonecrosis of the talus. Talus is the only bone of the lower limb to have more than 60% of its surface of cartilaginous component and has no muscle insertions; these characteristics are predisposing to its dislocation after trauma despite the stability provided by the tibio-talar joint.

Reviewing the literature, three authors reported cases of bilateral fracture-dislocation of the talus (Table I). Pogliacomi^1^ states that there are three points that should be taken into consideration: the surgical timing of treatment, the surgical approach and the stability of fixation. The patient with a fracture-dislocation of talus should be treated as soon as possible. In order to improve the exposure of the fracture site and reduce the dislocation, the osteotomy of the medial malleolus is necessary. The most common fixation method for fracture of the neck of the talus is the osteosynthesis with one or more screws. We think that, in case of fracture dislocation, the application of EF is necessary to prevent further soft tissue damage.

**Table I:** Bilateral Fracture-Dislocation of Talus: Review of Literature

Authors	Patient	Clinical condition	Treatment	Post-operative treatment	Patient condition at the last follow-up
Sayegh FE, *et al* (2009)3	29-year-old road traffic accident. Left intertrochanteric hip fracture, at right femoral shaft fracture, tibial shaft fracture.	Right: Comminuted fracture of the talus with subluxation of the ankle, subtalar and talonavicular joints Left: open fracture-dislocation Gustilo Type II of the talus. Open wound was at the dorso-lateral aspect of the joint.	Left: Irrigation with a total of 10L of normal saline with a pulsative lavage system. Debridement of devitalized soft tissue. Both: ORIF with K-wires.	Below knee non-weight bearing cast was applied for 8 weeks. Removal of cast and k- wires after 8 weeks.	At the 28-month: both ankle with signs of arthritis, no sign of AVN. ROM restricted: Plantar flexion 20°, dorsal flection 20°. Occasional discomfort but functional outcome satisfactory
Taraz-Jamshidi MH, *et al* (2013)4	25-year-old motor vehicle accident. T10, T11 vertebral body fractures.	Left ankle: Hawkin type 4 open talar neck fracture with subtalar and talonavicular dislocation. 3cm open wound. Right: Hawkin type 4 open and comminuted fracture of talar body and lateral process with subtalar and talonavicular dislocation. 3cm open wound	Right ankle: debridement Chevron Chevron osteotomy of medial malleolus. Reduction of talus fixed with 3 cancellous screws. Fixation of osteotomy with 2 malleolar pins. Left ankle: ORIF with antero medial and lateral approach. Fixation of neck fracture with 2 cannulated cancellous screws. Lateral malleolus fracture fixed with 2 pins.	Antibiotic prophylaxis continued up to 48 hours after the operation. Below knee non-weight bearing cast was applied.	At two years’ FU, AVN on the right side. ROM: right ankle with 20° of plantarflexion 0° of dorsiflexion with moderate pain. FADI 58/7. Left with 40° of plantarflexion 20° of dorsiflexion. FADI 82/7 SF-36 score: good quality of life.
Balaji GG, *et al* (2014)5	45-year-old, occupation accident, large stone follen. Bilateral femoral shaft fracture.	Right: open fracture dislocation, with anteromedial dislocation of the talus with the lateral process fracture. The wound of 10x4cm just below the medial malleolus with the talus lying completely outside the skin. The deficit of the toe flexion. Left: closed injury. Hawkin’s III type fracture dislocation of the talar neck	Right ankle: during debridement was found posterior tibial nerve stretched and posterior tibial artery lacerated. Reduction of talus fixed with External fixator. Left ankle: ORIF with medial malleolar osteotomy and fixation with 2 cannulated cancellous screws.	Left: Below knee non- weight bearing cast was applied. At 2 months the EF was removed and a below-knee splint was applied. At the end of the 4 months partial weight-bearing.	Left: the union of the fracture and sclerosis of the talar body. ROM: Right ankle with 15° of plantarflexion and 10° of dorsiflexion. Left ankle with 30° of plantarflexion and 10° of dorsiflexion. The patient returned to his job (carpenter) and walk up to 2km without of pain.
Silluzio N, *et al* (our case report)	29-year-old, aviator accident.	Right: fracture-dislocation Hawkin III Gustilo II. Left: Hawkin IV Gustilo IIIB	lavage with saline solution. Bilateral ORIF performing an osteotomy of the tibial malleolus and synthesis of the talus fractures with a 4.5mm cannulated screw and 2 screws for the synthesis of the osteotomy. The definitive stabilisation has been achieved in both limbs with an external fixator	At 2 months from the trauma, the EF was removed and applied an ankle brace (walker brace). The load with crutches and walker brace was granted after the control at the sixth month. Then we applied a bivalve-type brace	Radiograph shows a severe bilateral talonavicular arthrosis and moderate arthrosis of the sub-talus joint. The patient was satisfied with his quality of life and he returned to fly with his aeroplane.

The bilateralism of the fracture-dislocation of the talus is a rare occurrence and represents a challenging injury for orthopaedic surgeons. Proper treatment and correct timing are the key points to minimise the risk of complications and invalidating disability^1^. The use of external fixation, in association with ORIF of the fracture dislocation, is a good surgical solution for the stabilisation of Hawkin 3 and 4 fractures. Medial access through the osteotomy of the tibial malleolus could be necessary in order to obtain a good surgical exposure of the fracture site and a better reduction of the dislocation.
